# Candidate Multimodal MRI Markers of Persistent Auditory Verbal Hallucinations: A Controlled Pilot Study

**DOI:** 10.3390/tomography12080115

**Published:** 2026-08-18

**Authors:** Faten M. Aldhafeeri

**Affiliations:** Department of Health Information Management and Technology, College of Applied Medical Sciences, University of Hafr Al-Batin, Hafr Al-Batin P.O.Box 31991, Saudi Arabia; fmaldhafeeri@uhb.edu.sa

**Keywords:** auditory verbal hallucinations, cortical thinning, diffusion-tensor imaging, fractional anisotropy, functional MRI, structural MRI, white matter integrity

## Abstract

**Background/Objectives:** Auditory verbal hallucinations (AVH) are clinically heterogeneous experiences that may occur across psychiatric, neurological, sensory, and non-clinical contexts. This controlled pilot study investigated multimodal structural and functional MRI features associated with persistent AVH in a psychiatric clinical population, recruited from outpatient psychiatric clinics and diagnosed with schizophrenia, schizoaffective disorder, or bipolar disorder with psychotic features. Neurological, sensory, and non-clinical presentations of AVH were not included. **Methods:** This observational controlled pilot study included 14 participants with persistent auditory verbal hallucinations and 15 age- and sex-matched healthy controls. Participants with AVH had experienced the current persistent hallucinatory phase for a mean of 3.1 ± 1.6 years (range of 1–7), with a mean overall psychiatric illness duration of 12.4 ± 4.8 years. Independent component analysis assessed resting-state functional connectivity, BrainVoyager QX measured cortical thickness, and diffusion tensor imaging (DTI) evaluated white matter microstructure. Multiple comparisons were controlled using false discovery rate correction followed by 5000-iteration Monte Carlo cluster-extent correction for fMRI and Monte Carlo cluster correction for whole-brain structural metrics. **Results:** Participants with AVH demonstrated increased functional connectivity across default mode network (DMN) hubs (precuneus, inferior frontal, and parahippocampal gyri) and superior temporal regions. Whole-brain cortical thickness analysis revealed no significant group differences; however, secondary exploratory analyses of six regions previously implicated in AVH showed cortical thinning in participants with AVH relative to the controls after FDR correction. DTI revealed no group differences surviving whole-brain permutation correction (TFCE, FWE-corrected *p* < 0.05); exploratory uncorrected findings are reported as hypothesis-generating. **Conclusions**: This pilot study identifies structural and functional network differences between medicated individuals with persistent AVH and healthy controls, centred on frontotemporal and default mode networks. Because no psychiatric control group without AVH was included, these differences cannot be attributed specifically to AVH as opposed to the underlying psychiatric disorders or their treatment. No diffusion findings survived whole-brain permutation correction; exploratory uncorrected results are reported but are not incorporated into these conclusions. Collectively, these findings identify candidate imaging markers that require validation against psychiatric control groups.

## Simple Summary

People who hear voices showed stronger connections between brain regions involved in self-related thought and speech. When the whole brain was examined for differences in tissue thickness, none were found; however, when six specific regions previously linked to voice hearing in other studies were examined directly, these did show thinner tissue in areas linked to hearing and language. Some differences in white matter pathways were also observed, but these did not hold up when the analysis accounted for the very large number of comparisons made across the whole brain; so, they should be considered a preliminary lead rather than a confirmed finding. These findings are consistent with disrupted communication across multiple brain networks in people who hear voices, although the study could not separate effects of voice hearing itself from those of the underlying psychiatric conditions or their treatment.

## 1. Introduction

Auditory verbal hallucinations (AVH) are a multifaceted phenomenon observed in patients with psychiatric disorders. The experience of “hearing voices” without an objective external sound source has prompted considerable scientific investigation aimed at mapping its underlying neurobiological mechanisms [1]. AVH affect approximately 5–15% of the population worldwide [2], being reported in various clinical groups [3,4], as well as in healthy individuals [5]. Beyond schizophrenia, AVH are common comorbidities in several conditions, ranging from hearing impairment and epilepsy to cognitive-related disorders such as dementia and Parkinson’s or Alzheimer’s disease [6,7]. In addition, AVH have been observed in personality disorders, bipolar disorder, and major depressive disorder, underscoring their prevalence across a diverse array of psychiatric conditions [8].

Studies of neural correlates of AVH have revealed intricate interactions between different brain areas and networks, providing insights into the fundamental mechanisms underlying this perceptual experience. The main auditory regions, including Heschl’s gyrus, are involved in the development of AVH [9]. Furthermore, changes in the interhemispheric auditory pathway associated with the processing of auditory stimuli are thought to be pertinent to AVH pathophysiology [10]. Functional magnetic resonance imaging (fMRI) studies have identified abnormal connectivity patterns in people with AVH. Elevated functional connectivity within speech processing networks, such as the corticostriatal loop, has been linked to the development of AVH in patients with schizophrenia [11]. Furthermore, impairments in predictive coding have been postulated to underpin AVH in schizophrenia, resulting in resting hyperactivity in the sensory cortex [12]. Although much of the existing neuroimaging literature has focused on schizophrenia specifically, the present study examines persistent AVH across multiple psychiatric diagnostic categories within the psychotic spectrum—schizophrenia, schizoaffective disorder, and bipolar disorder with psychotic features—rather than restricting recruitment to a single diagnosis. This sample does not include AVH arising from neurological conditions (e.g., epilepsy, dementia, Parkinson’s disease), sensory deprivation, or non-clinical voice hearing.

Beyond functional connectivity findings, structural MRI studies have consistently implicated frontotemporal cortices in AVH. Voxel-based morphometry and cortical thickness work show grey-matter reductions and thinning in the left superior and middle temporal gyri (including Heschl’s/transverse temporal gyrus), often correlating with hallucination severity, and extending into inferior and middle frontal regions that subserve speech production and reality monitoring [13,14,15,16]. More recent structural analyses further corroborate abnormalities within temporal and prefrontal nodes among patients with a history or current presence of AVH, supporting a frontotemporal substrate for voice hearing [17].

Complementing these grey-matter effects, diffusion MRI studies indicate microstructural alterations in long-range language and auditory pathways. In particular, reduced integrity has been reported along interhemispheric auditory fibres and frontotemporal association tracts that link speech-perception and speech-production regions, consistent with a disconnection framework for AVH [18]. Taken together, prior structural and diffusion evidence converge with functional reports to implicate a distributed frontotemporal network—and its interhemispheric coupling—as a core substrate of AVH.

The disconnectivity of various resting-state networks has a significant impact on patients with schizophrenia, leading to AVH. Disrupted communication within these neural circuits, particularly involving the lateral prefrontal cortex (PFC) and the temporal and cingulate cortices, underpins the characteristic impairments in executive function, attention, language, and memory [19,20]. In a recent study, researchers explored the use of real-time fMRI neurofeedback targeting activity in the superior temporal gyrus of patients with schizophrenia and AVH, with promising results, suggesting that neurofeedback interventions could be a valuable tool for managing AVH [21].

Research examining metabolic activity during simulated auditory hallucinations in schizophrenia suggests that the development of AVH may be linked to dysfunctional modulation of the auditory cortex by limbic and thalamic circuits [22,23]. Furthermore, abnormal dynamic resting-state brain network organisation has been observed in patients with AVH, offering insights into the neurological processes driving these hallucinations [24]. Dynamic functional connectivity analyses have further revealed interhemispheric and intrahemispheric dysconnectivity in first-episode schizophrenia patients experiencing AVH [25]. In this study, we sought to investigate the neural correlates of AVH using structural and resting-state fMRI, with a specific focus on understanding the alterations in brain connectivity and structural integrity associated with this phenomenon.

In light of prior evidence implicating functional, structural, and diffusion abnormalities in AVH, this study was designed to test specific hypotheses across multiple neuroimaging modalities. First, we hypothesised that individuals with AVH would exhibit increased resting-state functional connectivity within auditory and self-referential networks, particularly involving the superior temporal and prefrontal cortices, compared with healthy controls. Second, we predicted that cortical thickness would be reduced in frontotemporal and cingulate regions implicated in speech processing and reality monitoring. Third, we expected diffusion tensor imaging to reveal diminished white matter integrity within frontotemporal association pathways and interhemispheric auditory fibres, consistent with a disconnection framework for AVH.

## 2. Materials and Methods

### 2.1. Study Participants

This study was approved by the Institutional Review Board of Hafr Al-Batin Central Hospital, Directorate of Health Affairs (protocol code: HPO00234/8; approved on 15 November 2023), and was conducted between January 2024 and December 2025. The study protocol strictly adhered to the ethical principles outlined in the Declaration of Helsinki, alongside all other applicable institutional regulations. Participants were recruited by convenience sampling from two outpatient psychiatric clinics between January 2024 and December 2025. Sixteen individuals with persistent AVH were assessed for eligibility; one was excluded prior to scanning following the incidental identification of an acoustic neuroma, and one was excluded after scanning owing to excessive head motion, which compromised data quality across all three modalities, yielding a final AVH sample of 14. Seventeen healthy volunteers were assessed for eligibility; two withdrew during the scanning session because of claustrophobia, yielding a final control sample of 15. Complete and usable data across all three imaging modalities were available for all 29 participants included in the analyses.

Participants were required to meet strict inclusion criteria—confirmed current AVH as the primary inclusion criterion, clinical stability, and MRI eligibility—which inherently constrained recruitment. Sample size was determined by the number of eligible participants available during the recruitment period rather than by an a priori power calculation. Although comparable group sizes are common in multimodal neuroimaging studies of AVH [13,17,18,26], we acknowledge that consistency with prior small studies does not establish statistical adequacy. A post hoc sensitivity analysis (two-tailed independent-samples *t*-test, α = 0.05, 80% power) indicated that this sample (*n* = 14/15) could reliably detect only large between-group effects (Cohen’s d ≥ 1.08). Precision of estimation is therefore emphasised through confidence intervals rather than post hoc power. Accordingly, the present investigation should be regarded as exploratory and hypothesis-generating rather than confirmatory. The sample provides adequate sensitivity only for large between-group effects, and smaller but potentially meaningful imaging differences may not have been detected. All findings therefore require replication in larger, independent cohorts before firm conclusions can be drawn.

Controls were frequency-matched to the AVH group at the group level on age and sex; individual pairwise matching was not performed, as reflected in the unequal group sizes (14 vs. 15). Between-group comparisons were therefore conducted using independent-samples tests, and no pairing was assumed in the statistical analysis. The controls were confirmed to have normal hearing by audiometric screening and screened negative for neurological or psychiatric illness using the SCID. SCID assessments were administered by a licensed psychiatrist affiliated with the outpatient psychiatric clinics from which AVH participants were recruited. Control SCID screening was conducted under blinded conditions, with the administering psychiatrist unaware of whether the individual being assessed was a prospective control or an AVH participant, reducing the risk of assessment bias. AVH participants were identified through referrals from outpatient psychiatric clinics and were clinically characterised as experiencing recurrent hallucinations for at least one year, with a minimum frequency of three episodes per week. The observed frequency was 18.5 ± 7.2 episodes per week (range of 3–35). Participants were confirmed to have no active psychotic episode at the time of data acquisition, as verified by the administering psychiatrist. Handedness was determined by self-report. Thirteen of 14 AVH participants (92.9%) and all 15 controls were right-handed; one AVH participant was left-handed. Handedness did not differ significantly between groups (Fisher’s exact test, *p* = 0.483). Smoking status was recorded by self-report. Eight of 14 AVH participants (57.1%) and 10 of 15 controls (66.7%) were current smokers; groups did not differ significantly (Fisher’s exact test, *p* = 0.710). Information on illness duration, AVH onset, and frequency was collected via clinical interviews. Age at first AVH onset refers to the age at which auditory verbal hallucinations were first experienced, whereas the duration of the current persistent AVH phase refers to the period of continuous, treatment-refractory hallucinations preceding scanning. These are reported separately, as participants had experienced hallucinations intermittently since illness onset. Participants were recruited transdiagnostically on the basis of a shared clinical presentation—persistent auditory verbal hallucinations—rather than a single categorical diagnosis. All diagnoses were established by a licensed psychiatrist using the Structured Clinical Interview for DSM-5 (SCID). The AVH cohort comprised individuals with schizophrenia (n=9), schizoaffective disorder (n=3), and bipolar disorder with psychotic features (n=2); The cohort therefore included participants across schizophrenia-spectrum and bipolar disorders who shared the clinical presentation of persistent AVH. Diagnosis was not modelled as a factor in the imaging analyses, both because the study was designed to identify neurobiological correlates converging across conditions and because the resulting subgroups (*n* = 2–9) were too small to support meaningful between-diagnosis comparison. Exclusion criteria included any history of major neurological conditions (e.g., epilepsy, dementia, Parkinson’s disease, traumatic brain injury), diagnosed hypertension or diabetes mellitus as documented in medical records, substance or alcohol use disorder, or MRI contraindications. These criteria were applied uniformly to both the AVH and control groups. All 14 AVH participants were receiving stable maintenance doses of atypical antipsychotics—olanzapine (*n* = 3), risperidone (*n* = 5), quetiapine (*n* = 3), and aripiprazole (*n* = 3)—for at least six weeks prior to scanning. The mean chlorpromazine-equivalent dose, calculated using the classical mean dose method [27], was 345.6 ± 112.4 mg/day (range of 200–600), and the mean duration of antipsychotic treatment was 4.2 ± 2.3 years. A subset of participants received adjunctive psychotropic medication: mood stabilisers (lamotrigine or carbamazepine; *n* = 2), antidepressants (*n* = 1), and benzodiazepines (*n* = 1). Adjunctive treatment was prescribed principally to participants with schizoaffective disorder or bipolar disorder with psychotic features, consistent with standard clinical management of these conditions.

All participants underwent pure-tone audiometry screening at standard octave frequencies (125, 250, 500, 1000, 2000, 4000, and 8000 Hz) to ensure normal hearing thresholds bilaterally; none exhibited hearing deficits. All structural and functional scans were visually inspected for quality and incidental findings by a single board-certified radiologist at the Central Hospital prior to analysis; formal inter-rater reliability was therefore not assessed.

### 2.2. Image Acquisition

A 32-channel head coil on a Siemens MAGNETOM Trio 3T whole-body MRI scanner (Siemens Healthineers, Erlangen, Germany) was used for data collection Resting-state fMRI parameters were as follows: echo-planar imaging (EPI) sequence with a 220 mm field of view (FOV), 3 mm slice thickness (3 × 3 × 3 mm^3^ voxel size), and 40 slices. We acquired 200 volumes with a repetition time (TR) of 2000 ms, echo time (TE) of 30 ms, and a flip angle of 90°. Participants were instructed to keep their eyes closed during acquisition, remaining awake and stationary throughout the scan.

For anatomical reference, 176 T1-weighted structural slices were acquired (FOV = 256 mm, 1 × 1 × 1 mm^3^ voxel size) using a magnetisation-prepared rapid gradient-echo sequence (TR = 2300 ms, TE = 2.98 ms, flip angle = 9°). The DTI acquisition followed a single-shell approach with 60 diffusion-weighted directions (b = 1000 s/mm^2^) and six b0 volumes, collected across 40 axial slices (no gap, 3 mm thickness, 3 × 3 × 3 mm^3^ voxel size) using a TR of 7500 ms and a TE of 115 ms.

### 2.3. fMRI Data Preprocessing and Analysis

BrainVoyager QX and Talairach-space normalization were used throughout for consistency with the ROI definitions and processing pipeline established in prior AVH neuroimaging work by this group [5], enabling direct methodological comparability across studies. Preprocessing of the neuroimaging data was performed using the BrainVoyager QX software package (v22.4; Brain Innovation, Maastricht, The Netherlands). Image preprocessing steps for the functional data included the application of temporal high-pass filters (using a 3-cycle cutoff), removal of linear trends, correction for 3D rigid-body movements, and slice-timing adjustments. Any participant scan demonstrating rotational movement beyond 2° or translational shifts greater than 3 mm was excluded from analysis. Following automated co-registration of functional and structural images, we performed spatial normalisation to the Talairach reference frame using a 12-parameter affine transformation. Normalised images were smoothed with an 8 mm full width at half maximum (FWHM) isotropic Gaussian kernel and resampled onto a 3 mm isometric grid.

Head motion was quantified for each participant as the mean framewise displacement across the resting-state run, computed from the six rigid-body realignment parameters as the sum of the absolute frame-to-frame changes in translation and rotation, with rotational displacements converted to millimetres assuming a 50 mm head radius. Mean framewise displacement did not differ significantly between groups (AVH: 0.18 ± 0.06 mm; controls: 0.15 ± 0.05 mm; t(27) = 1.25, *p* = 0.22), and no participant in the final analysed sample exceeded the exclusion thresholds specified above. One AVH participant was excluded prior to analysis on the basis of excessive head motion, as described in Section 2.1, and is not included in these figures. Volume censoring (scrubbing) was not applied. Nuisance regression of white matter, cerebrospinal fluid, or global signals was not performed. Because connectivity analyses were conducted within an independent component analysis (ICA) framework, these sources of variance, together with motion-related variance, are separated into distinct components rather than regressed from the timeseries: components exhibiting spatial and temporal signatures characteristic of head motion were identified during visual inspection and were not carried forward, with subsequent analyses restricted to the identified default mode network (DMN) component.

We employed the fastICA algorithm to perform ICA at both individual and group levels [28]. Thirty independent components were selected based on prior ICA studies of comparable resting-state datasets [29]. A self-organising grouped ICA analysis was then performed on all participants [30]. Component selection was performed on the group-level maps derived from all 29 participants combined, prior to any between-group comparison, and was therefore blind to group membership. Candidate resting-state networks were initially identified by inspection of the group-level maps, and the DMN component was then confirmed objectively by spatial template matching: the voxel-wise spatial correlation between each candidate component and the ten canonical resting-state network templates of Smith et al. [31] was computed. The selected component showed the highest correlation with the DMN template (r = 0.68) and was carried forward for subsequent analysis. Between-group comparisons of the DMN component were evaluated using a two-stage procedure. At the voxel level, statistical maps were thresholded using the Benjamini–Hochberg false discovery rate at q < 0.05, which corresponded to a realised uncorrected voxel-level threshold of *p* < 0.008. Surviving voxels were then subjected to cluster-extent correction using 5000 Monte Carlo simulations, which established a minimum cluster size of 200 contiguous voxels for a family-wise corrected cluster-level significance of α = 0.05 [32,33]. All reported clusters exceeded this extent threshold. Group comparisons were two-tailed independent-samples contrasts with 27 degrees of freedom, and exact corrected values for each cluster are reported in the results. Effect sizes for between-group comparisons were calculated as Cohen’s d using the pooled standard deviation, based on cluster-mean values extracted from each significant cluster.

### 2.4. DTI Data Preprocessing and Analysis

Motion artefacts and eddy current-induced distortions in the diffusion data were addressed via the FMRIB Diffusion Toolbox (FDT) within FSL v6.0. Non-brain tissues were stripped using FSL’s Brain Extraction Tool. DTI images were analysed using BrainVoyager QX (version 22.4) to estimate fractional anisotropy (FA) and mean diffusivity (MD). FA and MD maps were spatially normalised to anterior commissure–posterior commissure (ACPC) space and then to Talairach space. Voxel-wise statistical analysis of FA and MD maps, normalised to Talairach space within BrainVoyager, was performed using permutation-based non-parametric testing via the FSL *randomise* tool. To control the family-wise error (FWE) rate across the whole brain, Threshold-Free Cluster Enhancement (TFCE) was applied across 5000 permutations using the 3D volumetric option. Consistent with Section 2.6, no nuisance covariates were included in the imaging models, given the absence of significant between-group differences in age, sex, handedness, and smoking status. In addition to the fully corrected whole-brain analyses (FWE-corrected *p* < 0.05), uncorrected exploratory results (*p* < 0.005, minimum cluster extent of 100 contiguous voxels) are reported; we acknowledge that this extent criterion is a heuristic rather than a formally derived threshold, and that results at this threshold do not control the whole-brain false-positive rate.

### 2.5. Cortical Thickness Analysis

BrainVoyager QX software (version 22.4) was used for all cortical thickness analyses. T1-weighted images were corrected for field inhomogeneities to enhance brain segmentation. Anatomical data were converted to ACPC space and subsequently to Talairach standard space. Images were interpolated onto a 0.5 × 0.5 × 0.5 mm^3^ grid using sinc interpolation prior to segmentation. This upsampling does not increase the effective spatial resolution of the acquired 1 mm isotropic data; it provides a finer sampling grid that permits sub-voxel estimation of tissue boundaries during surface reconstruction and reduces discretisation error in the resulting thickness estimates. White/grey matter and grey matter/cerebrospinal fluid (CSF) boundaries were then derived using gradient-based binary segmentation. Segmentation and surface reconstruction were performed using BrainVoyager’s automated procedures; reconstructed surfaces were not subjected to systematic manual inspection or correction, and no automated quality-control metric was applied. This is acknowledged as a limitation (Section 4.2).

Because the whole-brain cortical thickness analysis revealed no significant group differences, we conducted a secondary analysis restricted to six cortical regions previously implicated in schizophrenia and AVH [17,34]. These regions comprised the right middle frontal gyrus, right anterior cingulate cortex, right middle temporal gyrus, right primary auditory cortex (Brodmann area 41), left posterior cingulate gyrus, and left superior temporal gyrus. Regions were defined using the Talairach Daemon-based anatomical atlas implemented in BrainVoyager QX v22.4, using gyral-level labels for five regions and the Brodmann-area definition for the primary auditory cortex; centre-of-gravity Talairach coordinates and volumes are provided in Table S1.

The predominance of right-hemisphere regions reflects the source literature: Zhao et al. [34] identified cortical thinning in right anterior cingulate cortex and right lateral temporal cortex in first-episode psychosis, right Heschl’s gyrus in the pooled analysis, and bilateral lateral middle temporal cortex with age-related progression. The right middle frontal gyrus and left posterior cingulate gyrus were included on theoretical rather than meta-analytic grounds, reflecting the role of dorsolateral prefrontal cortex in reality monitoring and of the posterior cingulate as a core DMN hub.

Although the direction and general anatomical focus of these comparisons followed the a priori hypothesis stated in the Introduction, the specific region set was selected from the published literature after the whole-brain analysis had been examined. These analyses are therefore secondary and exploratory rather than confirmatory and are interpreted accordingly throughout.

### 2.6. Statistical Analysis

Outcomes. The primary outcome was between-group difference in resting-state functional connectivity within the default mode network. Secondary outcomes were between-group differences in cortical thickness across six a priori regions of interest, and in fractional anisotropy and mean diffusivity. Demographic and clinical comparisons were treated as descriptive.

Models and contrasts. All imaging comparisons were two-group, two-tailed independent-samples contrasts between AVH participants and the controls, evaluated in both directions (AVH > controls and controls > AVH). Functional connectivity was assessed by group-level independent component analysis with random-effects comparison of the identified DMN component. Cortical thickness was compared vertex-wise at the whole-brain level and subsequently within literature-derived ROIs. Diffusion measures were compared voxel-wise for FA and MD separately. Demographic variables were compared using independent-samples *t*-tests for continuous measures and Fisher’s exact test for categorical measures.

Covariates. No covariates were included in the imaging models. Groups did not differ significantly in age, sex, handedness, or smoking status, and the available sample was insufficient to support reliable covariate adjustment. Residual confounding by unmeasured variables cannot be excluded (Section 4.2).

Correction for multiple comparisons. Functional connectivity maps were thresholded at the voxel level using the Benjamini–Hochberg false discovery rate (q < 0.05; realised threshold *p* < 0.008), followed by cluster-extent correction using 5000 Monte Carlo simulations, yielding a minimum cluster size of 200 voxels at a corrected cluster-level α = 0.05. Whole-brain cortical thickness analyses used Monte Carlo cluster correction; ROI-level analyses were corrected across the literature-derived ROIs using Benjamini–Hochberg FDR, with q < 0.05 considered significant. Diffusion analyses were first evaluated with whole-brain FDR correction (*p* < 0.05); as no clusters survived, results are reported at an uncorrected threshold of *p* < 0.005 with a minimum cluster size of 100 voxels and are designated exploratory.

Effect sizes. Cohen’s d was calculated using the pooled standard deviation, with 95% confidence intervals derived using the Hedges–Olkin normal approximation.

Missing data and outliers. Complete data were available for all 29 participants included in the analyses; no imputation was required. No formal outlier screening was undertaken, and no participant was excluded on the basis of extreme values.

Assumption checks. Formal tests of normality and homogeneity of variance, such as the Shapiro–Wilk and Levene’s tests, were not performed. Such tests have limited power at this sample size, and parametric analyses were applied on the assumption of approximate normality. This is acknowledged as a limitation (Section 4.2).

Software. Imaging preprocessing and analysis were performed in BrainVoyager QX v22.4 (Brain Innovation, Maastricht, The Netherlands). Diffusion preprocessing used the FMRIB Diffusion Toolbox and Brain Extraction Tool within FSL v6.0, with white matter clusters annotated using the JHU-ICBM-DTI atlas. Demographic comparisons and effect-size calculations were performed in IBM SPSS Statistics (version 27; IBM Corp., Armonk, NY, USA).

## 3. Results

Table 1 provides a detailed summary of participant demographics.

**Figure 1:**
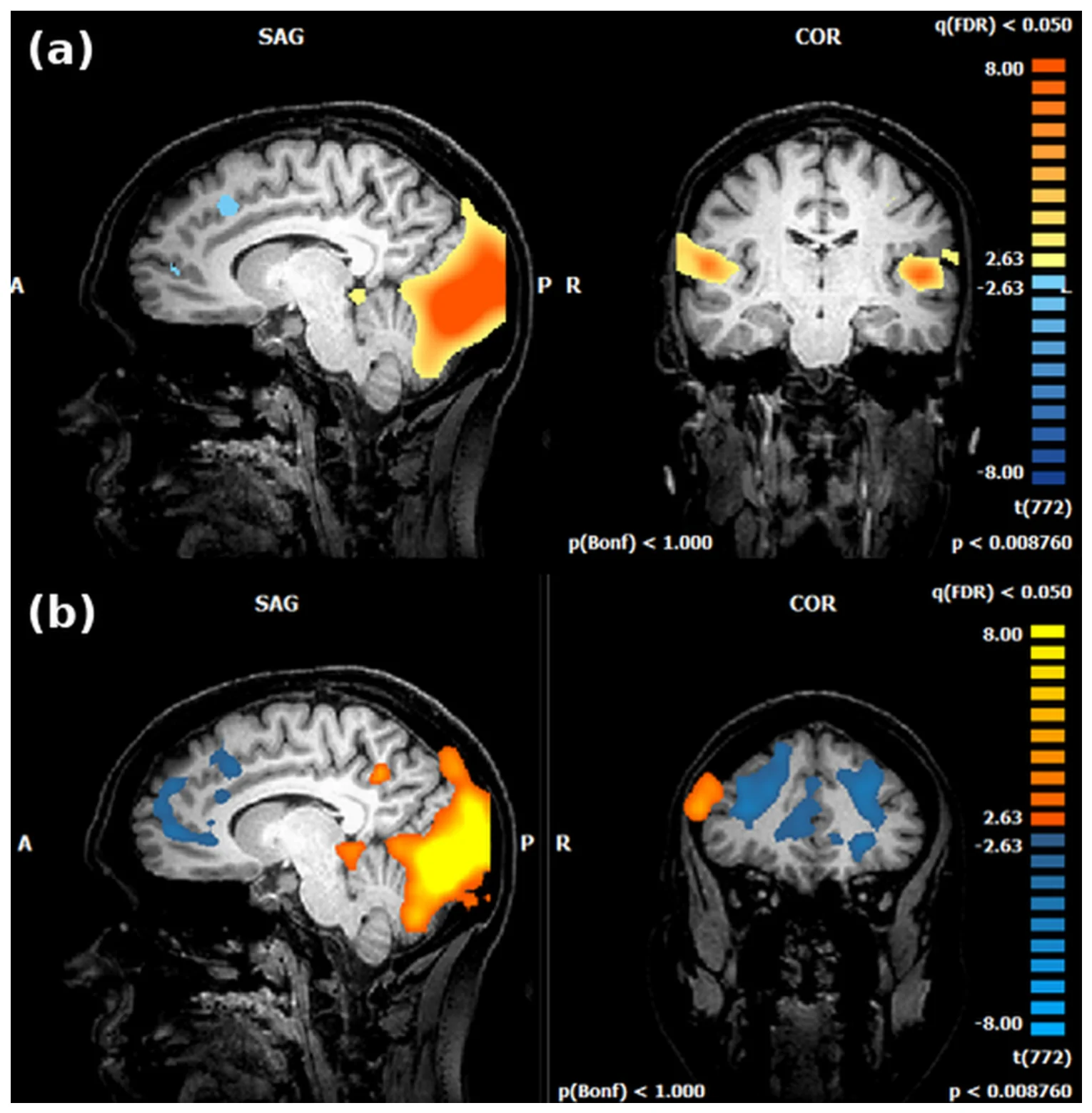
Comparison of functional connectivity within the default mode networks in individuals with AVH and matched controls (voxel-level q < 0.05, FDR-corrected; minimum cluster extent 200 voxels, Monte Carlo corrected at α = 0.05). ((**a**), upper panel) Orange voxels indicate regions where participants with AVH exhibited greater functional connectivity, including canonical DMN nodes such as the precuneus, inferior frontal gyrus, and superior/middle temporal gyri. ((**b**), lower panel) Blue voxels indicate regions of relatively lower connectivity in the AVH group. Images were generated using BrainVoyager QX software (version 22.4; Brain Innovation, Maastricht, The Netherlands). SAG, sagittal view; COR, coronal view; p(Bonf), Bonferroni-corrected significance threshold; q(FDR), false discovery rate–corrected threshold; t(772) denotes the t-statistic with 772 degrees of freedom shown on the colour bar. Orientation labels indicate anatomical direction: A, anterior; P, posterior; R, right hemisphere.

A significantly enhanced connectivity was revealed within the DMN in individuals with AVH compared with the controls (Figure 1a). Increased connectivity was observed in canonical DMN hubs (precuneus, inferior frontal gyrus, parahippocampal gyrus), as well as in lateral temporal regions including the bilateral superior and middle temporal gyri. While the superior temporal gyrus (STG) is not a core DMN hub, its involvement suggests functional interactions between auditory/speech networks and DMN-related processes in individuals with AVH. Functional connectivity was lower in the AVH group than in controls between the left inferior frontal gyrus, superior frontal gyri (bilaterally), and left anterior cingulate (Figure 1b; Table 2).

**Figure 2:**
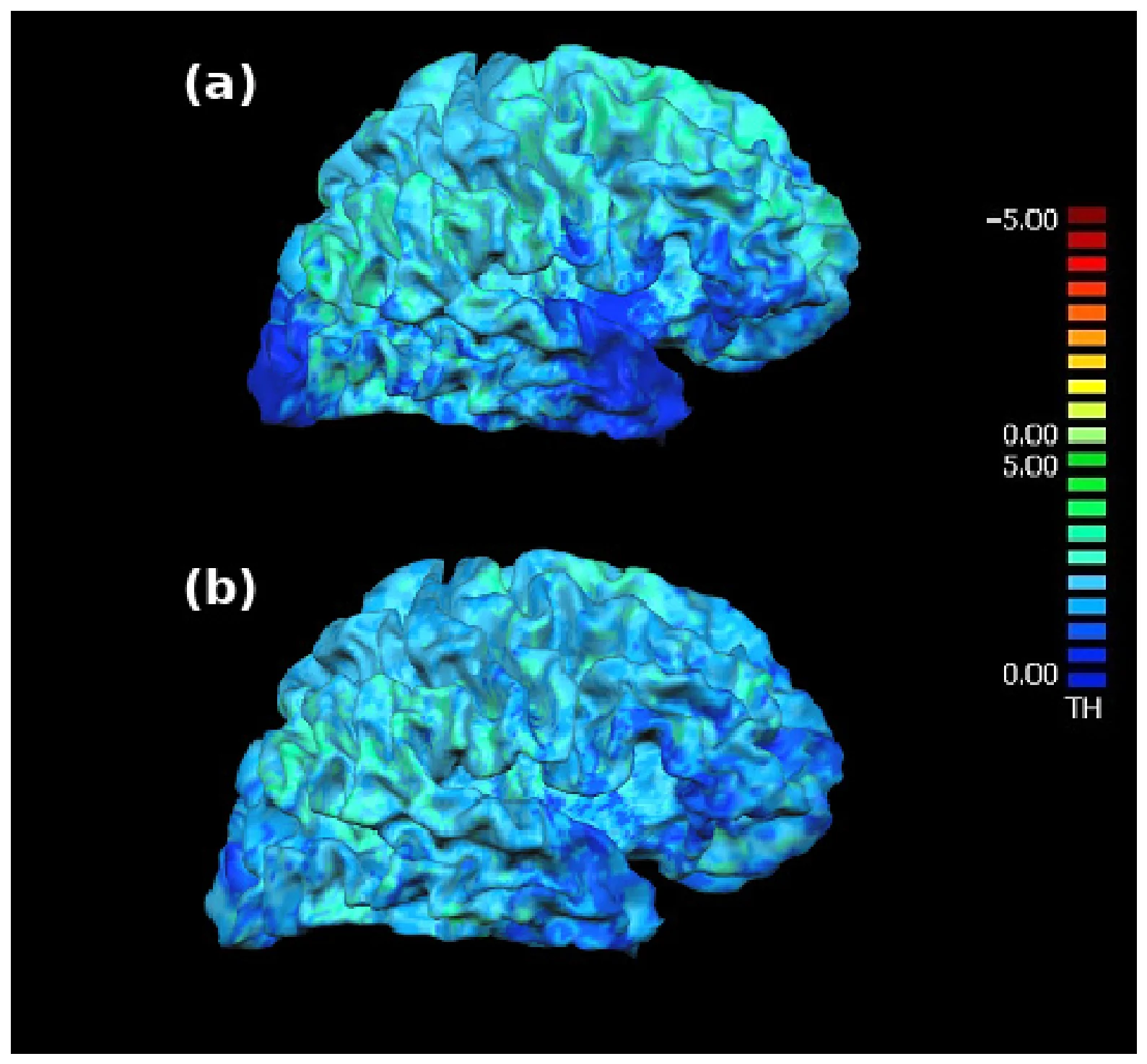
Cortical thickness maps. The average cortical thickness is shown on the group-averaged brain for participants with AVH (**a**) and healthy controls (**b**). The scale on the right shows thickness (TH) measured in millimetres. Images were generated using BrainVoyager QX software (version 22.4; Brain Innovation, Maastricht, The Netherlands). Colours represent cortical thickness in millimetres according to the scale (TH) on the right, with warmer colours indicating greater thickness and cooler colours indicating lesser thickness.

Whole-brain cortical thickness analysis using FDR correction (*p* < 0.05) revealed no significant differences between groups. In secondary, exploratory ROI analyses, cortical thinning was observed in six literature-derived regions in participants with AVH relative to healthy controls, with mean differences ranging from 0.20 to 0.42 mm and large to very large effect sizes (Cohen’s d = 0.80–1.52). After Benjamini–Hochberg FDR correction across the six literature-derived ROIs, all six regions remained significant (Figure 2; Table 3). The precision of these estimates varied appreciably: the right anterior cingulate cortex (d = 0.83, 95% CI 0.07–1.59) and right middle temporal gyrus (d = 0.80, 95% CI 0.04–1.56) had intervals extending close to zero, and these two effects should be interpreted with particular caution. By contrast, the largest effects—left superior temporal gyrus (d = 1.52, 95% CI 0.69–2.35) and right primary auditory cortex BA 41 (d = 1.33, 95% CI 0.53–2.14)—have lower bounds well above zero, indicating more precisely estimated effects.

Values are group means ± SD. Cohen’s d calculated using pooled SD. Confidence intervals for Cohen’s d were calculated using the Hedges–Olkin normal approximation. Cohen’s d is reported as an absolute magnitude; the direction of each effect is indicated by the sign of the mean difference, with negative values denoting cortical thinning in the AVH group. *p*-values are derived from Welch’s independent-samples *t*-test. FDR-adjusted q-values were calculated using the Benjamini–Hochberg procedure across the six literature-derived ROIs. Results with q < 0.05 were considered significant after ROI-level FDR correction. Negative mean differences indicate cortical thinning in the AVH group relative to the controls. R = right hemisphere; L = left hemisphere; BA 41 = Brodmann Area 41. Whole-brain FDR-corrected analysis revealed no significant group differences in cortical thickness. ROI-level analyses were corrected across the six literature-derived ROIs using Benjamini–Hochberg FDR, and all six ROI effects remained significant after ROI-level FDR correction. All findings remain preliminary given the pilot sample and warrant replication.

**Figure 3:**
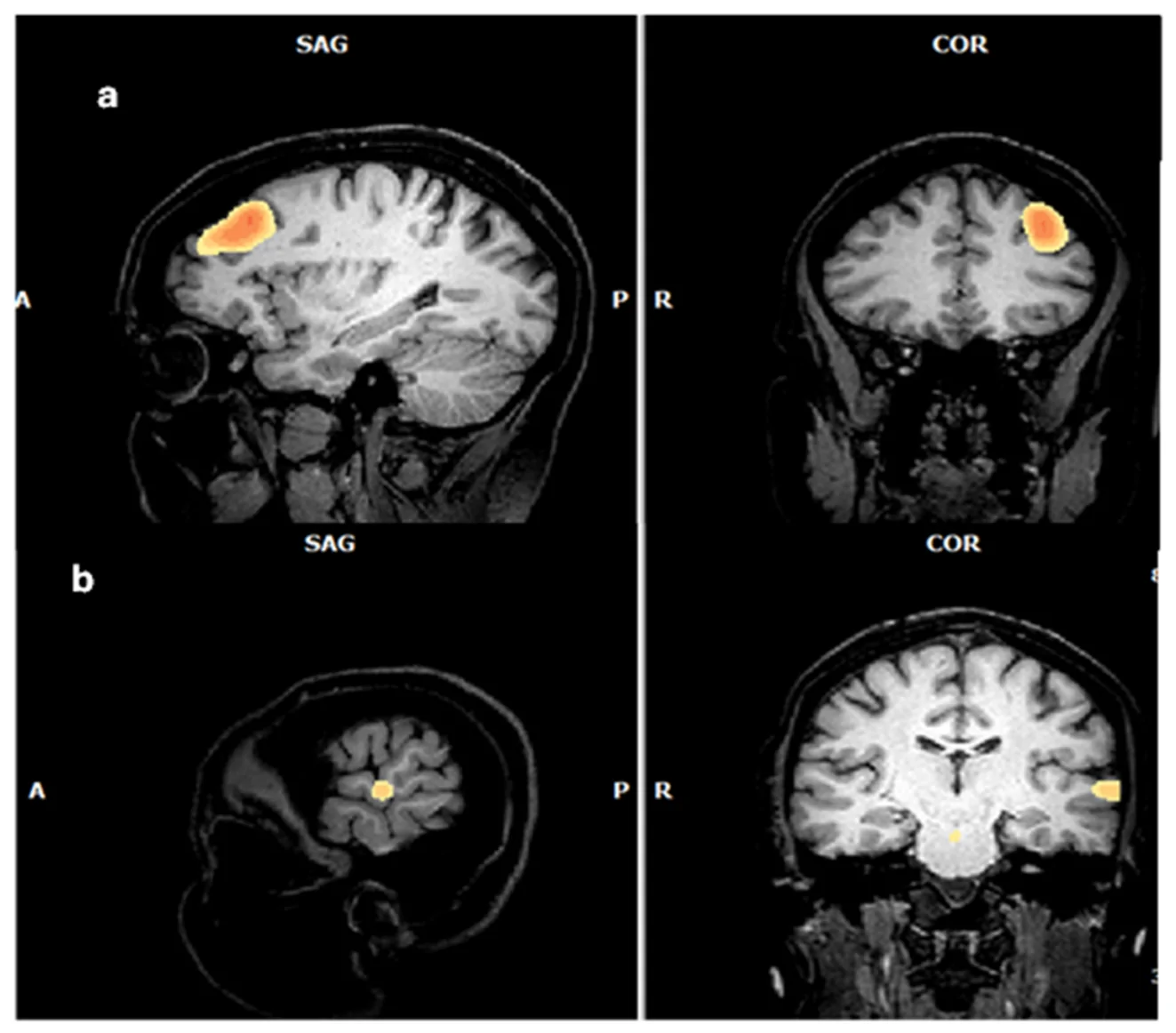
Diffusion tensor imaging findings in participants with AVH relative to controls, at a statistical threshold of *p* < 0.005 (uncorrected for multiple comparisons) and a minimum cluster size of 100 contiguous voxels. (**a**) Reduced fractional anisotropy (FA) in the left middle frontal gyrus in individuals with AVH compared with controls (controls > AVH). (**b**) Increased mean diffusivity (MD) in the left superior temporal gyrus in individuals with AVH relative to controls (AVH > controls). Images were generated using BrainVoyager QX software (version 22.4; Brain Innovation, Maastricht, The Netherlands). SAG, sagittal view; COR, coronal view. Orientation labels indicate anatomical direction: A, anterior; P, posterior; R, right hemisphere.

Whole-brain voxel-wise permutation correction (TFCE, 5000 iterations) revealed no significant clusters surviving family-wise error (FWE) correction at *p* < 0.05. At an exploratory uncorrected threshold (*p* < 0.005, minimum cluster size 100 voxels), reduced FA was observed in participants with AVH relative to the controls in the left middle frontal gyrus (135 voxels; Talairach −26, 32, 28; JHU-ICBM label: anterior thalamic radiation; mean FA 0.41 ± 0.05 versus 0.48 ± 0.06; t(27) = −3.42, *p* = 0.002, d = 1.26). Increased MD was observed in the left superior temporal gyrus (112 voxels; Talairach −42, −24, 4; JHU-ICBM label: superior longitudinal fasciculus; AVH 0.79 ± 0.04 × 10^−3^ mm^2^/s versus controls 0.74 ± 0.03 × 10^−3^ mm^2^/s; t(27) = 3.68, *p* = 0.001, d = 1.42). Because these clusters did not survive whole-brain permutation correction, they are classified strictly as exploratory and are not incorporated into the study’s conclusions (Table 4; Figure 3).

## 4. Discussion

The temporal cortex, particularly the superior temporal gyrus, plays a central role in AVH, with both structural and functional involvement. Mechelli et al. demonstrated that impaired functional integration between the superior temporal and anterior cingulate cortices was linked to false auditory perception in patients with AVH [35], a deficit that likely contributes to the failure to recognise self-produced vocalisations as internally generated rather than external [36]. This aligns with evidence that left-hemisphere language-region activation during AVH may reflect misrepresentation of internally generated speech [37]. Consistent with prior literature [38], the present study likewise observed alterations in canonical DMN hubs, including the precuneus and frontotemporal regions, supporting a model in which temporal cortex involvement in AVH extends beyond localised structural change to broader network-level dysconnectivity.

Reality-monitoring deficits, implicating both the PFC and ACC, offer a further explanatory framework for AVH. Kawaguchi et al. reported that reduced mismatch negativity amplitude at left frontal electrodes correlated with hallucination severity, indicating impaired neural differentiation between self-generated and external speech [39], while individuals with AVH are more likely to confuse imagined and heard words, underscoring a broader failure of source monitoring [40]. The ACC, implicated in self-monitoring and error detection, is typically deactivated prior to AVH onset, a shift in cognitive state that may predispose individuals to misattribute internally generated speech as external [41,42,43]. Together, these findings suggest that impaired cognitive control across frontal and cingulate regions contributes to the characteristic misattribution of self-generated auditory experience in AVH.

The parahippocampal gyrus (PHG), associated with memory processing and contextual retrieval, is activated during AVH, consistent with a role in retrieving auditory memories during conscious perception [37]. Abnormalities in the PHG may contribute to hallucinations by supplying missing auditory information, as proposed by Bayesian brain accounts [44], and increased PHG connectivity with auditory processing regions has been reported in AVH, suggesting network-level rather than purely localised disruption [45].

Collectively, these findings implicate temporal, prefrontal, cingulate, and parahippocampal regions in a distributed disconnectivity model of AVH, with evidence from structural, functional, and diffusion measures (Table 2, Table 3 and Table 4; Figure 3).

### 4.1. Comparison with Previous Multimodal Investigations of Persistent AVH

The present findings converge most clearly with prior work at the level of functional connectivity. Using a comparable group-ICA approach in a similarly small sample, Wolf et al. reported disrupted connectivity of temporal and cingulate cortices in patients with persistent AVH, characterised by reduced cingulate connectivity alongside increased connectivity in bilateral temporal regions relative to the controls [19]. This pattern is closely mirrored in the present data, in which AVH participants showed increased connectivity in bilateral superior and middle temporal regions together with reduced connectivity in the left anterior cingulate, suggesting that this specific direction of temporo-cingulate dysconnectivity may be a relatively replicable feature of persistent AVH across independent samples.

At the structural level, the present cortical thinning findings partially overlap with those of Sone et al., who reported reduced surface area in the left caudal middle frontal gyrus and precentral gyrus among patients with a history of AVH [17]. Both studies therefore implicate frontal motor and premotor cortex in AVH, although the present findings were right-lateralised and concerned cortical thickness rather than surface area—a structurally and genetically distinct component of cortical morphology. The convergence in general anatomical territory, despite divergence in laterality and morphometric measure, is consistent with the possibility that frontal regions are affected through more than one structural pathway in AVH.

The diffusion findings are more difficult to reconcile with earlier work. The present study found reduced FA in the anterior thalamic radiation and increased MD in the superior longitudinal fasciculus, whereas the seminal work of Hubl et al. reported increased FA in the temporoparietal arcuate fasciculus and anterior corpus callosum in individuals with AVH [26]. This divergence may reflect differences in sample composition (transdiagnostic and antipsychotic-treated in the present study versus a smaller, medication-heterogeneous schizophrenia sample), tract segmentation approach, or the exploratory, uncorrected threshold applied here. It may also reflect genuine biological heterogeneity: reports of both increased and decreased FA across a range of tracts appear throughout the schizophrenia and AVH literature, including reduced cingulum bundle integrity [46] in addition to the perisylvian and frontotemporal findings discussed above. The present findings should therefore be interpreted as contributing one further data point to this still-unresolved picture rather than as a definitive characterisation of white matter involvement in AVH.

Taken together, these comparisons suggest that convergent evidence across independent multimodal studies is currently the strongest for functional dysconnectivity involving temporal and cingulate regions, while structural and particularly diffusion findings remain more variable across studies and require replication before firm anatomical conclusions can be drawn.

### 4.2. Study Limitations

Several limitations of this study warrant consideration. First, the absence of a psychiatric control group without AVH represents a fundamental design limitation. All participants with AVH carried a diagnosis of a schizophrenia-spectrum or bipolar disorder and were receiving antipsychotic treatment; so, the observed differences relative to healthy controls cannot be attributed specifically to AVH rather than to the underlying psychiatric disorder, its broader symptomatology, or its pharmacological treatment. The findings should therefore be interpreted as differences between medicated individuals with persistent AVH and healthy controls, and the regions identified regarded as candidate markers requiring validation. Establishing AVH specificity will require a three-group design comparing individuals with AVH, diagnosis-matched patients without AVH, and healthy controls.

Second, the relatively small sample size (14 AVH participants, 15 healthy controls; total N = 29) represents a notable limitation. Recruitment was constrained by the strict eligibility criteria necessary for clinical and neuroimaging validity—participants were required to have confirmed current AVH, maintain clinical stability, and be free of MRI contraindications—limiting the pool of eligible participants within the study setting during the 2024–2025 recruitment period. Although this sample size aligns with that of prior neuroimaging studies of AVH and was sensitive only to large between-group effects, it may have been insufficient to detect small-to-moderate neurobiological differences at the whole-brain level under stringent multiple-comparison correction, likely accounting for the absence of FDR-significant cortical thickness differences in the whole-brain analysis despite meaningful findings in the exploratory ROI analyses. The modest sample also limits generalisability to broader or more heterogeneous populations and precludes meaningful subgroup analyses by medication type, illness duration, or AVH severity.

Third, although the integration of multimodal neuroimaging techniques—structural MRI, resting-state fMRI, and DTI—represents a methodological strength, variability in imaging acquisition parameters and preprocessing pipelines across sites and studies may affect the reproducibility and comparability of results. In addition, the use of Talairach-space normalization and BrainVoyager QX, while internally consistent, differs from the Montreal Neurological Institute (MNI)-based pipelines (e.g., fMRIPrep, FreeSurfer, ANTs) now widely adopted in the field; replication using contemporary normalization frameworks would strengthen cross-study comparability. Unlike the fMRI and cortical thickness analyses, which were corrected for multiple comparisons (FDR and Monte Carlo cluster correction, respectively), DTI findings were reported at an uncorrected threshold of *p* < 0.005. This decision reflects both methodological considerations and sample-size constraints, as applying strict whole-brain correction to diffusion analyses in a small sample would substantially reduce statistical power and risk masking genuinely relevant white matter abnormalities. Accordingly, the DTI findings should be regarded as exploratory and interpreted with caution, pending replication in larger, independent samples.

Fourth, formal assumption testing was not undertaken, and parametric analyses were applied on the assumption of approximate normality; at this sample size, tests of normality and homogeneity of variance have limited power to detect violations. The ROI analyses were secondary and were specified after the whole-brain analysis had been examined; they should therefore be regarded as exploratory and require confirmation in an independent sample. Two of the six regions were included on theoretical rather than meta-analytic grounds. In addition, cortical surface reconstructions were generated using automated segmentation without systematic visual inspection or manual correction. Undetected segmentation errors, particularly at the grey matter–CSF boundary, may therefore have contributed to variability in the thickness estimates. Similarly, all scans were visually inspected for quality and incidental findings by a single board-certified radiologist, and formal inter-rater reliability was not assessed. The absence of a second independent rater means that undetected rating variability cannot be excluded, although this procedure was applied uniformly across both groups. Hypertension and diabetes mellitus were excluded on the basis of documented medical history rather than direct screening at the time of scanning (e.g., blood pressure measurement or glycaemic testing), and undiagnosed or subclinical vascular or metabolic conditions cannot be fully excluded.

Fifth, although recruitment was transdiagnostic with respect to categorical diagnosis, all participants met criteria for a schizophrenia-spectrum or bipolar disorder, and the majority carried a diagnosis of schizophrenia. The present findings therefore cannot be generalised to AVH arising in other contexts, including sensory impairment, neurodegenerative disease, epilepsy, or non-clinical voice hearing, all of which may involve distinct neurobiological mechanisms. Subgroup sizes further precluded any assessment of whether the observed imaging patterns differ across the diagnoses represented.

Sixth, psychotropic polypharmacy represents a further limitation. Beyond antipsychotic treatment, a subset of participants received mood stabilisers, antidepressants, or benzodiazepines. The structural effects of the mood stabilisers used in this cohort are not well characterised, and their potential contribution to the observed cortical thickness differences cannot be quantified. The functional findings are less susceptible to this concern, as benzodiazepines typically attenuate resting-state connectivity, opposing rather than mimicking the hyperconnectivity observed in the AVH group. The sample was too small to model medication class as a covariate, and residual pharmacological effects cannot be excluded.

Seventh, the cross-sectional design of this study precludes any inference regarding the temporal dynamics of the observed structural and functional abnormalities or their relationship to clinical trajectory.

Eighth, clinical characterisation was limited to variables recorded at the time of scanning. No standardised AVH severity instrument, such as the PSYRATS or PANSS, was administered; hallucinations were characterised by frequency and duration only. This precluded correlation of imaging measures with symptom severity, and future studies should incorporate validated severity ratings to permit brain–symptom association analyses. Comorbid psychiatric diagnoses beyond the primary diagnosis were not systematically recorded, and educational attainment was likewise unavailable; neither could therefore be examined as a potential confounder. Smoking status did not differ significantly between groups; notably, current smoking was somewhat more prevalent among the controls than among AVH participants, making it unlikely that the cortical thinning observed in the AVH group reflects smoking-related effects.

## 5. Conclusions

This multimodal neuroimaging study identifies structural and functional differences between medicated individuals with persistent AVH and healthy controls. Participants with AVH exhibited hyperconnectivity within canonical DMN hubs and auditory-DMN interfaces, and cortical thinning in key auditory and prefrontal ROIs (Cohen’s d = 0.80–1.52). Diffusion analyses revealed no group differences surviving whole-brain permutation correction; exploratory findings obtained at an uncorrected threshold are reported in the results section but are not incorporated into these conclusions. Together, these findings are consistent with a multimodal disconnectivity model of persistent AVH in medicated psychiatric populations and support further validation in larger, clinically characterised cohorts.

Future research should aim to address the limitations of this study by employing larger, multisite cohorts to improve statistical power and generalisability. Longitudinal designs would be particularly valuable for examining how connectivity patterns and structural abnormalities evolve over the course of illness and in response to treatment. A further direction lies in multimodal data fusion and machine-learning approaches capable of jointly modelling functional, structural, and diffusion biomarkers, rather than analysing each modality in isolation as in the present study. Techniques such as linked independent component analysis and multiset canonical correlation analysis with joint ICA can identify covarying patterns across modalities that univariate, modality-specific analyses cannot detect, while supervised classification could eventually support individual-level characterisation or prediction of AVH severity and treatment response. Realising this potential will require sample sizes substantially larger than the present cohort, as such models typically demand far more observations than predictors to achieve stable and generalisable performance. Neuromodulatory approaches to AVH are not solely prospective: repetitive transcranial magnetic stimulation targeting temporoparietal cortex has been investigated clinically for over two decades, and real-time fMRI neurofeedback targeting the superior temporal gyrus has shown preliminary evidence of symptom reduction in small trials [21]. Whether the specific frontotemporal and DMN circuits implicated in the present study represent useful refinements to these existing targets remains an open question for future investigation. These preliminary group-level associations may help to inform the design of future studies examining the neural correlates of persistent AVH in larger and more comprehensively characterised samples.

## Figures and Tables

**Table 1:** Demographic and clinical characteristics.

Characteristic	AVH Group (*n* = 14)	Control Group (*n* = 15)	*p*-Value
Age (years), mean ± SD (range)	48.3 ± 5.7 (40–57)	46.6 ± 4.3 (41–60)	0.370
Sex, *n* (male/female)	9/5	8/7	0.710
Handedness, *n* right-handed (%)	13 (92.9)	15 (100)	0.483
Current smokers, *n* (%)	8 (57.1)	10 (66.7)	0.710
Diagnosis, *n*	Schizophrenia, 9; schizoaffective disorder, 3; bipolar disorder with psychotic features, 2	—	—
Psychiatric illness duration (years), mean ± SD (range)	12.4 ± 4.8 (6–22)	—	—
Duration of current persistent AVH phase (years), mean ± SD (range)	3.1 ± 1.6 (1–7)	—	—
Age at first AVH onset (years), mean ± SD (range)	36.2 ± 4.5 (33–45)	—	—
AVH frequency (episodes/week), mean ± SD (range)	18.5 ± 7.2 (3–35)	—	—
Antipsychotic class	Atypical (all participants)	—	—
Antipsychotic agent, *n*	Olanzapine, 3; risperidone, 5; quetiapine, 3; aripiprazole, 3	—	—
Chlorpromazine-equivalent dose (mg/day), mean ± SD (range)	345.6 ± 112.4 (200–600)	—	—
Antipsychotic treatment duration (years), mean ± SD (range)	4.2 ± 2.3 (3–9)	—	—
Adjunctive psychotropic medication, *n*	Mood stabilisers (lamotrigine or carbamazepine), 2; antidepressants, 1; benzodiazepines, 1	—	—

AVH = auditory verbal hallucinations; SD = standard deviation. Values are presented as mean ± SD (range) or *n* (%), as indicated. *p*-values refer to between-group comparisons; age was compared using an independent-samples *t*-test, and sex, handedness, and smoking status using Fisher’s exact test. Chlorpromazine-equivalent doses were calculated using the classical mean dose method [27]. All AVH participants were receiving stable maintenance doses of atypical antipsychotics for at least six weeks prior to scanning. Adjunctive psychotropic medication was prescribed principally to participants with schizoaffective disorder or bipolar disorder with psychotic features. Em dashes (—) indicate variables not applicable to the control group. Diagnostic inclusion criteria and exclusion criteria, including major neurological conditions, hypertension, diabetes mellitus, and substance or alcohol use disorder, are detailed in Section 2.1.

**Table 2:** Comparison of functional connectivity of brain regions within the DMN in participants with AVH and matched controls.

Region	TAL (x, y, z)	AVH Mean ± SD	HC Mean ± SD	T Value	Voxels	Cohen’s d	95% CI for d	*p*-Value	FDR q-Value
AVH > HC									
R. inferior frontal gyrus	41, 13, 24	0.58 ± 0.22	0.12 ± 0.15	7.20	617	2.46	1.49, 3.42	<0.001	<0.001
R. superior temporal gyrus	49, −6, 3	0.21 ± 0.19	−0.05 ± 0.18	3.36	436	1.41	0.59, 2.22	0.002	0.008
R. middle temporal gyrus (BA 21)	55, −3, −11	0.38 ± 0.20	0.02 ± 0.16	4.64	798	2.00	1.10, 2.89	<0.001	<0.001
R. precentral gyrus	41, −3, 34	0.62 ± 0.21	0.08 ± 0.14	8.27	854	3.05	1.98, 4.12	<0.001	<0.001
L. parahippocampal gyrus (BA 36)	−34, −29, −19	0.24 ± 0.18	−0.10 ± 0.19	4.15	388	1.84	0.97, 2.70	<0.001	0.002
L. superior temporal gyrus (BA 22)	−49, −17, 4	0.54 ± 0.21	0.05 ± 0.17	6.85	788	2.57	1.59, 3.56	<0.001	<0.001
HC > AVH									
R. superior frontal gyrus	19, 42, 30	0.11 ± 0.16	0.45 ± 0.19	−3.97	523	1.93	1.05, 2.81	<0.001	0.003
L. anterior cingulate	−10, 37, 0	0.10 ± 0.17	0.38 ± 0.20	−3.36	446	1.50	0.68, 2.33	0.002	0.008
L. medial frontal gyrus (BA 10)	−23, 37, 25	0.09 ± 0.17	0.42 ± 0.18	−3.89	305	1.88	1.01, 2.76	<0.001	0.004
L. superior frontal gyrus (BA 9)	−17, 47, 28	0.14 ± 0.17	0.51 ± 0.21	−4.28	567	1.93	1.05, 2.81	<0.001	0.002

AVH = auditory verbal hallucinations; HC = healthy controls; TAL = Talairach coordinates of the cluster peak; R = right hemisphere; L = left hemisphere; BA = Brodmann area; SD = standard deviation; CI = confidence interval; DMN = default mode network. FDR = false discovery rate, a correction for multiple comparisons (Benjamini–Hochberg procedure) that controls the expected proportion of false positives among the clusters declared significant, thereby limiting Type I error across the whole-brain analysis. Group means represent cluster-mean connectivity values (z-transformed) extracted from each significant cluster. Confidence intervals for Cohen’s d were calculated using the Hedges–Olkin normal approximation. Cohen’s d is reported as an absolute magnitude; the direction of each effect is indicated by the sign of the mean difference or T value. As clusters were defined on the basis of the same group contrast, these estimates are not independent of the selection procedure and should be interpreted as descriptive indices of effect magnitude rather than unbiased estimates. FDR correction was applied at the voxel level across the whole brain within the DMN component mask, as described in Section 2.3.

**Table 3:** Comparisons of cortical thickness between AVH participants and controls in six literature-derived ROIs (secondary exploratory analysis, with effect sizes and 95% confidence intervals).

Region	AVH (*n* = 14) Mean ± SD (mm)	Controls (*n* = 15) Mean ± SD (mm)	Mean Diff (mm)	Cohen’s d	95% CI for d	*p*-Value	FDR q-Value	Interpretation
Middle frontal gyrus (R)	2.55 ± 0.26	2.89 ± 0.28	−0.34	1.26	0.46, 2.05	0.002	0.003	Exploratory; FDR-significant
Anterior cingulate (R)	2.65 ± 0.22	2.85 ± 0.26	−0.20	0.83	0.07, 1.59	0.034	0.041	Exploratory; FDR-significant
Middle temporal (R)	2.96 ± 0.31	3.20 ± 0.29	−0.24	0.80	0.04, 1.56	0.041	0.041	Exploratory; FDR-significant
Primary auditory cortex BA 41 (R)	2.50 ± 0.33	2.90 ± 0.27	−0.40	1.33	0.53, 2.14	0.002	0.003	Exploratory; FDR-significant
Posterior cingulate gyrus (L)	2.90 ± 0.32	3.30 ± 0.31	−0.40	1.27	0.47, 2.07	0.002	0.003	Exploratory; FDR-significant
Superior temporal gyrus (L)	2.85 ± 0.26	3.27 ± 0.29	−0.42	1.52	0.69, 2.35	< 0.001	0.003	Exploratory; FDR-significant

**Table 4:** Diffusion tensor imaging findings in participants with AVH relative to controls at the exploratory uncorrected threshold (*p* < 0.005, minimum cluster size 100 voxels).

Measure	Region	JHU-ICBM Label	TAL (x, y, z)	Cluster Size (Voxels)	AVH Mean ± SD	HC Mean ± SD	T Value	*p* (Uncorrected)	Cohen’s d	95% CI for d
FA	Left middle frontal gyrus	Anterior thalamic radiation	−26, 32, 28	135	0.41 ± 0.05	0.48 ± 0.06	−3.42	0.002	1.26	0.47, 2.06
MD	Left superior temporal gyrus	Superior longitudinal fasciculus	−42, −24, 4	112	0.79 ± 0.04	0.74 ± 0.03	3.68	0.001	1.42	0.61, 2.24

AVH = auditory verbal hallucinations; HC = healthy controls; FA = fractional anisotropy; MD = mean diffusivity; TAL = Talairach coordinates of the cluster peak; JHU-ICBM = Johns Hopkins University International Consortium for Brain Mapping white matter atlas; SD = standard deviation; CI = confidence interval. FA is dimensionless; MD values are expressed as1 ×10^−3^ mm^2^/s. Confidence intervals for Cohen’s d were calculated using the Hedges–Olkin normal approximation. Cohen’s d is reported as an absolute magnitude; the direction of each effect is indicated by the sign of the mean difference or T value. Whole-brain permutation correction (TFCE, 5000 permutations) revealed no clusters surviving family-wise error correction at *p* < 0.05.

## Data Availability

The data supporting the findings of this study are available from the corresponding author upon reasonable request. Restriction is necessary because the dataset comprises structural and functional MRI scans linked to individual psychiatric diagnoses, medication history, and demographic information from a small clinical sample (*n* = 29) recruited from two identifiable outpatient clinics.

## Supplementary Materials

The following supporting information can be downloaded at: https://www.mdpi.com/article/10.3390/tomography12080115/s1, Table S1: Definitions of the six cortical regions of interest used in the secondary cortical thickness analysis.
