# Supplementary material for: Candidate Multimodal MRI Markers of Persistent Auditory Verbal Hallucinations: A Controlled Pilot Study
Source: Tomography. 2026 Aug 18;12(8):115. doi: 10.3390/tomography12080115 (PMC13517848; doi:10.3390/tomography12080115)
Supplement: Supplementary file 1 [file tomography-12-00115-s001.zip › tomography-4445327-supplementary.pdf]

Table S1. Definitions of the six cortical regions of interest used in the secondary cortical thickness analysis.

Region of interest	TAL (x, y, z)	Volume (mm³)	Literature source
Middle frontal gyrus (R)	32, 24, 30	356	Reality-monitoring rationale (see Section 2.5)
Anterior cingulate cortex (R)	6, 30, 18	411	[32]
Middle temporal gyrus (R)	54, −28, −10	608	[32]
Primary auditory cortex, BA 41 (R)	48, −22, 8	398	[32]
Posterior cingulate gyrus (L)	−8, −48, 22	311	Core DMN hub (see Section 2.5)
Superior temporal gyrus (L)	−52, −18, 4	299	[16,32]

R = right hemisphere; L = left hemisphere; TAL = Talairach coordinates of the region centre of gravity; BA = Brodmann area; DMN = default mode network. Regions were defined using the Talairach Daemon–based anatomical atlas implemented in BrainVoyager QX v22.4 (Brain Innovation, Maastricht, Netherlands), using gyral-level labels for five regions and the Brodmann-area definition for the primary auditory cortex. Four regions were derived from published cortical thickness findings in schizophrenia and AVH; the right middle frontal gyrus and left posterior cingulate gyrus were included on theoretical grounds, reflecting their roles in reality monitoring and as a core DMN hub respectively. Regions were selected following the non-significant whole-brain analysis; the resulting comparisons are therefore secondary and exploratory rather than confirmatory (see Section 2.5).
